# Practice Guidelines for Canadian Neurophysiology Laboratories During the COVID-19 Pandemic

**DOI:** 10.1017/cjn.2020.184

**Published:** 2020-08-19

**Authors:** Juan Pablo Appendino, Steven K. Baker, Kristine M. Chapman, Tamara Dykstra, Tabrez Hussein, Michelle-Lee Jones, Michelle M. Mezei, Seyed M. Mirsattari, Marcus Ng, Joanne Nikkel, Vaso Obradovic, Cecile Phan, Lawrence Robinson, Angela Scott, Jose Tellez-Zenteno, Michelle Van Niekerk, Shannon Venance, Fraser Moore

**Affiliations:** Pediatric Neurology, Alberta Children’s Hospital, Calgary, AB, Canada; University of Calgary, Calgary, AB, Canada; Department of Medicine, Physical Medicine & Neurology, Peripheral Nerve Clinic, McMaster University, Hamilton, ON, Canada; Division of Neurology, Department of Medicine, University of British Columbia, St. Pauls, Canada; Vancouver Hospital, Vancouver, BC, Canada; EMG Clinic: Day Hospital, Riverview Health Centre, One Morley Avenue, Winnipeg, MB, Canada; Diagnostic Neurophysiology, BC Children’s Hospital, Vancouver, BC, Canada; Max Rady College of Medicine, Rady Faculty of Health Sciences, University of Manitoba, Winnipeg, MB, Canada; Division of Neurology, Department of Medicine, Faculty of Medicine, University of British Columbia, Vancouver General Hospital, Vancouver, BC, Canada; Departments of Clinical Neurological Sciences, Medical Imaging, Medical Biophysics and Psychology, Western University, London, ON, Canada; University of Manitoba, Winnipeg, MB, Canada; Neurodiagnostics Department, Health Sciences Center, Winnipeg, MB, Canada; Supervisor Neuromuscular Diseases Unit-VGH, Diamond Health Care Center, Vancouver, BC, Canada; Division of Neurology Faculty, Department of Medicine, Faculty of Medicine & Dentistry, University of Alberta, Edmonton, AB, Canada; Physical Medicine and Rehabilitation, University of Toronto, Toronto, ON, Canada; St. Joseph’s Health Care London, London, ON, Canada; University of Saskatchewan, Saskatchewan Epilepsy Program, Division of Neurology, Department of Medicine, Royal University Hospital, Saskatoon, SK, Canada; Aviva Medical Specialists Clinic Inc., Barrie, ON, Canada; Department of Clinical Neurological Sciences, Schulich School of Medicine & Dentistry, Western University, London, ON, Canada; Department of Neurology and Neurosurgery, McGill University, Montréal, QC, Canada

**Keywords:** Neurophysiology, Laboratory, Practice, Safe, Guidelines, Clinical, COVID-19, Canada, SARS-CoV-2, EMG, EEG, EMU, IOM, EP

## Abstract

The COVID-19 pandemic has had a major impact on clinical practice. Safe standards of practice are essential to protect health care workers while still allowing them to provide good care. The Canadian Society of Clinical Neurophysiologists, the Canadian Association of Electroneurophysiology Technologists, the Association of Electromyography Technologists of Canada, the Board of Registration of Electromyography Technologists of Canada, and the Canadian Board of Registration of Electroencephalograph Technologists have combined to review current published literature about safe practices for neurophysiology laboratories. Herein, we present the results of our review and provide our expert opinion regarding the safe practice of neurophysiology during the COVID-19 pandemic in Canada.

## RÉSUMÉ

**Lignes directrices et pratiques exemplaires destinées, dans le contexte de la pandémie de COVID-19, aux laboratoires canadiens de neurophysiologie.** La pandémie de COVID-19 a eu un impact majeur sur la pratique clinique. En matière de pratique, des normes sécuritaires demeurent justement essentielles afin de protéger les travailleurs de la santé tout en leur permettant de prodiguer des soins adéquats. La Société canadienne des neurophysiologistes cliniques (CSCN), l’Association canadienne des technologues en électro-neurophysiologie, l’Association canadienne des technologues en électromyographie, le *Board of Registration of Electromyography Technologists of Canada* et le *Conseil canadien d’enregistrement des technologues en électroencéphalographe* se sont ainsi associés afin de passer en revue la littérature scientifique actuelle portant sur les pratiques sécuritaires au sein des laboratoires de neurophysiologie. Nous souhaitons donc présenter ici les résultats de notre revue de littérature et proposer, dans le contexte de la COVID-19, un regard éclairé en ce qui regarde les pratiques sécuritaires en neurophysiologie au Canada.

## Introduction

Given that the current COVID-19 pandemic will continue to affect Canadian health care for the foreseeable future, our organizations felt that it was appropriate to provide some guidance on safe practices during the pandemic. We begin by presenting the key points in table form in order to emphasize the practical aim of this article. The main body of the article elaborates on these points and provides the scientific rationale for their inclusion, when this exists.

## Purpose

To provide guidelines for safe functioning of neurophysiology laboratories during the COVID-19 pandemic, recommendations are based on expert opinion and review of relevant published guidelines. These guidelines are not intended to replace institutional, regional, or provincial protocols.

## Background

Named “Severe Acute Respiratory Syndrome (SARS)-CoV-2” by the Coronaviridae Study Group, the coronavirus causing the current pandemic is responsible for the third outbreak of acute respiratory syndrome in the last two decades after the SARS in 2003 and the Middle East Respiratory Syndrome (MERS) in 2012.^20^ The disease caused by this coronavirus is known as “COVID-19” and the full spectrum of its symptomatology and pathophysiology is yet to be defined. As its name implies, the respiratory symptoms (cough, fever, and dyspnea) are present in more than 60% of hospitalized patients and are the main cause of death.^21^ It is clear that staff in the neurophysiology laboratory will be exposed to COVID-19 due to the nature of this disease. The reported neurological manifestations include a wide range of symptoms and signs such as headaches, anosmia and ageusia, impaired consciousness, toxic-metabolic encephalopathy, encephalitis, seizures and status epilepticus, strokes and vascular events, Guillain–Barré syndrome, myositis, and peripheral nerve disorders.^22^

The current ongoing Canadian provincial plans to re-launch the economy are at different levels of complexity in each province. However, some have already announced the re-opening of regulated health profession services and some have scheduled non-urgent surgeries as long as they continue to follow approved guidelines set by their professional colleges.^23^

These developments have led the Canadian Society of Clinical Neurophysiologists (CSCN), the Canadian Association of Electroneurophysiology Technologists, the Association of Electromyography Technologists of Canada, the Board of Registration of Electromyography Technologists of Canada, and the Canadian Board of Registration of Electroencephalograph Technologists to elaborate this guideline for safe practice in the neurophysiology laboratories of Canada.

## General Infection Control Recommendations (Table 1)

Booking of patients for neurophysiology testing should be prudent and minimize unnecessary testing. If the test will not change clinical management, avoid an emergency department visit, or avoid morbidity and mortality it should in general be deferred. During crisis or peak periods of the pandemic, only cases that are urgent or time-sensitive should be seen, such as those posing threat to life, limb, or long-term outcome.^1,2^ As the pandemic starts to come under control and laboratories gain greater experience with safely seeing patients, the threshold for selecting appropriate patients for neurophysiological testing will become progressively lower.^3–6^ Patient workflow should still be adjusted in order to assess high-risk cases safely.^1,3^ In COVID-19-positive cases, neurodiagnostic testing should be performed only in the inpatient setting with full personal protective equipment (PPE) equipment as per local protocols, only if test results will change management (treatment and/or diagnosis), and ideally in an airborne infection isolation room (AIIR) as the virus could survive several hours to days on certain surfaces.^2,4,7^ If done in AIIR, these tests should be postponed for at least 4 h if recent aerosol generating medical procedure (AGMP) had been performed (i.e., nebulizer therapy, chest physiotherapy, intubation, bronchoscopy, sputum induction, bilevel positive airway pressure [BiPAP], or continuous positive airway pressure [CPAP] use).^5,7^ The outpatient setting is reserved for COVID-19-negative patients when triaging deems necessary.^3-6,8^

**Table 1:** General infection control recommendations^1–13^

Scheduling should be guided by medical urgency, laboratory/staff limitations, and waiting room occupancy considerations (respecting 2-m physical distancing). All patients should undergo screening for COVID-19 exposure by questionnaire or patient transport service checklist. If local institutional versions do not exist, online versions are available. Clinical neurophysiological investigations require prolonged, close contact with patients. All patients and companions, doctors, and technologists should be required to wear a mask and gloves unless otherwise contraindicated. Testing of COVID-19-positive patients should be performed only if critical for patient diagnosis and medical management. In the training laboratory, the number of personnel should be minimized. One instructor and one trainee are preferable. Accompanying escorts/caregivers should be accommodated only if essential, with close adherence to distancing requirements. Use of PPE and precautions consistent with institutional or other conventionally accepted guidelines for known or suspected COVID-19-positive patients must be applied. Staff must complete PPE use training and use PPE consistent with institutional/provincial guidelines. Whenever possible, disposable equipment should be used. All exposed non-disposable neurodiagnostic equipment should be sanitized between patients with facility approved disinfectant preparations according to manufacturer’s recommendations. Consider dedicating portable units for use in high-risk areas and/or for positive/probable cases of COVID-19

The waiting room must be utilized in a way that will allow a 2-m gap (physical distancing) between patients, including not concomitant bookings if needed due to limited physical space.^1-4,9^ Electroencephalogram (EEG) and electromyogram/nerve conduction study (EMG/NCS) technologists must avoid remaining in the waiting room to minimize physical contact with patients. There should be sufficient time between appointments to allow for distancing arrangements and the cleaning of equipment.^2,4^ If multiple clinics running simultaneously share the waiting room, staff should consider the implications of a large volume of patients in shared spaces, including corridors.^1^ Special attention should be paid to patients requiring escorts (i.e., pediatric or with disabilities). These patients’ requisitions should be flagged in order to be identified in advance and account for the extra space needed in the waiting room. Prohibiting visitors and family members from attending the visit unless necessary is encouraged. The use of a clinical and epidemiological screening tool looking for high-risk patients prior to confirming the appointment and right before proceeding with the test (if done on a different date) is recommended.^1,3,4^ Everyone, including all health care workers (HCWs), patients, and visitors, should be considered as potential carriers of COVID-19 in order to minimize risks.^10,11^

Laboratory staff must be screened with a fit for work screening tool or questionnaire on a regular basis, preferable on a daily basis, to prevent institutional spreading of the infection.^1,10,11^ They should adhere to all guidelines for physical distancing, isolation, or quarantine if required. Appropriate precautions, as dictated by local infection prevention and control (IPAC) leaders, should be used at all times at work.^11^ Considerations for limiting technologist exposure to high-risk patients include limiting tests to only as long as absolutely necessary for a clinical decision, reducing inpatient technologist coverage hours, implementing alternate technologist shifts, identifying experienced technologists to manage challenging cases, and delaying testing until such expertise is available (although caution may be required for technologists over 60 years of age), considering mitigating factors (i.e., pregnancy, immunodeficiency, and living with elders), and providing resources for technologist counselling.^1-4,6^ Laboratory leaders should reinforce that appropriate use of recommended equipment and processes will protect staff. They should also minimize personal displays of fear or anxiety that are not productive or helpful.^2^

Re-triaging and prioritization of all cancelled or postponed patients is important including updates of the current status on their clinical conditions to confirm whether the test is still necessary or if an upgraded triage score is needed. Inpatients and after hours requests should be triaged by the attending neurologist on service or on-call – not a designate. Triaging of outpatient requests should be done by the laboratory director or a designate approved by the laboratory director. Patients must have appropriate notification on file and on the test requisition of confirmed or suspected COVID-19 infection to alert laboratory staff.^2-4^

PPE occupies a key role in preventing infection and dissemination of the disease. Three different tiers of facial masks are available including respirator masks (N95 and FFP2 variants) with higher filtering capacity particularly in aerosol-generating procedures. The second tier include the surgical masks which are classified into three levels according to their filtration efficacy. They offer protection against droplets from direct spatter although they are not as effective as the previous category for small particles. There is a variant of these masks which includes eye plastic shield increasing the protection capabilities. Finally, single-use masks may be made of a single layer and are not used in the healthcare setting as they can control mainly large volume of saliva emission. Two other elements for facial protection are available. Facial shields provide an additional physical barrier and could be utilized along with the previously mentioned masks to minimize risks. Powered air-purifying respirators are mainly used in dentistry practice. The last two options should be considered for high-risk procedures.^12,13^ Surgical facial masks (preferable with plastic eye shield) and gloves should be used by the patient, escorts, technologists, physicians, and trainees at all times during the duration of the encounter. In select circumstances, N95 masks may be used if deemed appropriate (i.e., single-fiber EMG [SFEMG] in facial muscles and confirmed COVID-19-positive patient).^2,4,9^ Disposable or reusable PPE and biohazard waste management must follow well-known recommendations (i.e., Government of Canada, Center of Disease Control). Please refer to their websites for further details as it is beyond the scope of this document.^10,11^ Staff should be trained in PPE use.^2^

Equipment and testing rooms must be cleaned, disinfected, and sanitized at the beginning of the working day and after every patient tested. Minimal handling of equipment and testing materials is also desirable in order to minimize the possibilities of contamination. Consider the use of dedicated NCS/EMG/EEG/evoked potential (EP) equipment for high-risk areas and/or positive/probable cases with COVID-19.^2,7^ Strict cleaning procedures, that follow local IPAC guidance, should include but are not limited to^1–8^:

- Cleaning all surfaces patients touch (beds, linens, chairs, doorknobs, grab bars, etc.)
- Cleaning all surfaces staff touch (keyboards, carts, cables, etc.)
- Disposable items should be used whenever cleaning is not feasible.
- If not disposable, consider covering ancillary equipment pieces in disposable single-use clear medical grade plastic, especially if equipment (e.g., jackboxes) is located close to the patient.
- Consider consulting the manufacturers of NCS/EMG/EEG/EP units to ensure surface disinfectants used are compatible with the external hardware of the units and do not pose a risk of compromising equipment.
- The EEG equipment will usually require cleaning of the amplifier (headbox), computer – keyboard, cables – wires, particularly if these are non-disposable. Extra time is needed for the disinfectant to air dry. This procedure usually takes extra 15–30 min per patient of the regular 75–90 min appointment.
- Cleaning of an EMG/NCS equipment may be quicker as most items making contact with the patient are disposable. An additional 15 min for cleaning/disinfecting should suffice.

Clinicians may want to consider providing some part of the care via virtual platforms such as initial history taking, discussion of results, and/or follow-up visits.

## EMG/NCS Specific Recommendations (Table 2)

In general, EMG/NCS in the setting of COVID-19 requires focus on the prevention of spread through contact and droplets, in addition to the usual body fluid precautions. Unless there is an AGMP in process (e.g., a patient is intubated, or on CPAP, or BiPAP,) or facial SFEMG is performed, there is no need for N95 masks and eyes protection.^2-4^ Although performing facial SFEMG is not considered an aerosolization maneuver, due to the proximity to involuntary sources of rapid exhalation (i.e., cough and sneeze) and the length of time exposed to the patient, a preference for using an N95 mask and facial shield or goggles seems reasonable based on individual risk assessment at the time.^2,4,8,9^ Surgical masks (preferable with plastic eye shield) should be considered when performing peripheral EMG/NCS in COVID-19-negative patients; however, in COVID-19-positive patients, due to the potential aerosolization of the virus in the room, N95 mask and facial shield or goggles should be used.^9,12,13^ Testing should be performed as many hours after an AGMP was performed as possible (preferable after 4 h).^6,7^ Both patient and HCWs should have PPE appropriate for the prolonged, close contact.^10,11^

**Table 2:** EMG/NCS specific recommendations^2–4,6–13^

Testing should be the minimum requirement for diagnosis and management including the number of conduction studies and/or the number of muscles assessed with needle EMG. When time-sensitive interventions or diagnostic accuracy is hinging upon EMG/NCS, these should be prioritized and performed using appropriate precautions.^8^ Examples include (but are not limited to) patients waiting for nerve transfers or those with suspected Guillain–Barré syndrome, myasthenia gravis, or amyotrophic lateral sclerosis. Surgical mask and facial shield or goggles are considered satisfactory for the majority of EMG procedures. Although performing facial SFEMG is not considered an aerosolization maneuver, due to the proximity to involuntary sources of rapid exhalation (i.e., cough and sneeze) and the length of time exposed to the patient, a preference for using an N95 mask and facial shield or goggles seems reasonable based on individual risk assessment at the time.

Testing should be the minimum requirement for diagnosis and management. This will minimize in person contact. Measures should be taken to reduce the risk of patient-to-patient transmission such as doing inpatient studies on the wards instead of bringing patients to the lab.^2,4^

When time-sensitive interventions or diagnostic accuracy is hinging upon EMG/NCS, these should be prioritized and performed using appropriate precautions.^8^ Examples of such situations include patients who may be candidates for nerve transfers, patients with rapidly progressive deficits (e.g., suspected Guillain–Barré syndrome), patients in whom definitive diagnosis is required for treatment (e.g., generalized myasthenia gravis when the wait for serology results could be prolonged), or patients waiting for confirmation of serious conditions (e.g., suspected amyotrophic lateral sclerosis). When there is uncertainty about the urgency of a particular referral, then consideration should be given to contacting the referring physician and obtaining further information.

## EEG and EMU Specific Recommendations (Table 3)

The decision to use collodion versus paste and tape must consider possible aerosolization risk with collodion application against the need to reapply paste and tape electrodes from poor adherence.^2,4,6^ The length of the study should be short enough to address the patient management and at the same time to minimize the exposure of the technologist to a high-risk patient maintaining the minimal standards.^4,5^ Hyperventilation should not be routinely performed, but if justified for high diagnostic yield (e.g., pediatric absence epilepsy), patients must wear surgical masks.^4-6^

**Table 3:** EEG, neurostimulation, and EP specific recommendations^2–7,9,12^

For electrode application, the decision to use collodion versus paste and tape must consider possible aerosolization risk with collodion application against the need to reapply paste and tape electrodes due to poor adherence. The test’s length should be sufficient to address patient management while maintaining minimal standards. Hyperventilation should not be routinely performed. If justified for high diagnostic yield (e.g., pediatric absence epilepsy), patients must wear surgical masks. In the inpatient setting, equipment should be outside the patient’s room using a long cable if possible. Stimulation for reactivity assessment can still be performed. Nasal/oropharyngeal swab test for rapid COVID-19 detection could be considered at admission to EMU with the aim to mitigate contagious risks. EMU admissions should consider whether a single companion is allowed in the EMU when needed for safety and/or diagnostic accuracy. Priority indications may include but are not limited to: potential for management changes based on monitoring, pre-surgical workup (phase I, II, and ictal SPECT), high seizure burden, SUDEP risk, threat of disease worsening, and frequent emergency department visits. If ambulatory EEG is used as an EMU alternative it should include video, ideally with outpatient dis/connection. VNS/DBS insertion for epilepsy, setting revisions, or battery exchange should be considered only if seizure frequency and severity outweighs COVID-19 risk. Cleaning of EP equipment must use disinfectants that are compatible, and if using earbuds or inner ear electrodes for BAEP, they must be discarded after each use.

SUDEP = sudden unexpected death in epilepsy; SPECT = single-photon emission computed tomography.

Utilization of surgical mask and goggles or N95 and facial shield should be considered for inpatients when in the ward or intensive care units (ICUs), respectively. In COVID-19-positive patients, the second facial protection combination is preferred.^7,9,12,13^ Inpatient/continuous EEG should situate the portable video-EEG system outside the patient room using long cables if safe and necessary, particularly if COVID-19 positive or if the physical space in the epilepsy monitoring unit (EMU) room does not allow to place the EEG machine 2 m apart from the patient.^4^ The number of different technologists entering each patient room should be limited. Stimulation for reactivity assessment can still be performed. Recordings should last at least 20 min, but may be delayed by at least 4 h to reduce potential airborne transmission if there has been nebulizer treatment.^2,4,6^

Prior to EMU reopening, ensure adequate staffing and rescue medications, mask and COVID-19 testing availability, limiting patient breaks, available ICU beds for post-operative care and status epilepticus, and physical distance from COVID-19 areas. Rounds and surgical conferences should be virtualized, with consideration to additional operations and business meetings to review lists of previously cancelled/held admissions, new admissions, and intracranial subdural/stereo-EEG/electrocorticography logistics.^4,14^ Admissions should consider whether nasal/oropharyngeal swab for rapid COVID-19 testing is deemed necessary as an early infection detection could prevent further contagious disease.^15^ Consideration should be present for allowing family members in the EMU if needed for safety and diagnostic accuracy. Priority indications may include potential for management changes based on monitoring, pre-surgical workup (phase I, II, and ictal single-photon emission computed tomography), high seizure burden, sudden unexpected death in epilepsy risk, threat of disease worsening, and frequent emergency department visits.^14^ To avoid cross-contamination between hospital populations, EMUs should be populated by persons with, or suspected to have, epilepsy. EMUs may also consider initially reopening at half capacity with physical distancing and enhanced barrier protection measures (e.g., plastic over curtains).^2,4,14^

As a possible EMU alternative, ambulatory EEG should include video, ideally with outpatient dis/connection external to hospital, plastic covering for community COVID-19 settings, and allowing a “rest period” between uses for potentially difficult to disinfect ambulatory pieces (e.g., pouches and straps), while keeping in mind battery limitations.^4-6,14^

## Neurostimulation (Table 3)

If stable, consider using virtual visits and deferring programmatic changes, with alternatives such as optimizing anti-seizure medications and instituting a rescue medication strategy. While vagus nerve stimulator (VNS) auto-titration and deep brain stimulation (DBS) program sets allow automated changes, decompensation may occur in remote settings, and surgical procedures may be limited even in urban centres. The need for in-person programming/evaluation should consider factors such as status epilepticus, increased seizures, seizure-related injuries, severe stimulation-related side effects, battery depletion, non-superficial hardware infection, and hardware malfunction on a case-by-case basis.^16^

## EP Specific Recommendations (Table 3)

Consult the manufacturer of goggles and headphones to ensure surface disinfectants used are compatible with the external hardware and do not pose a risk of compromising equipment or patient care. If using earbuds or inner ear electrodes for sound generation, they must be discarded after each use.^4^ When somatosensory EPs are needed in the ICU setting, assess whether the patient poses a risk of aerosol-generating procedures and ensure adequate PPE is used according to your facilities’ recommendations; N95 mask may be necessary in these cases.^2,4,7^

## IOM Specific Recommendations

Patients with active infectious disease, including COVID-19, or those on droplet or contact precautions are unlikely to undergo surgery requiring intraoperative monitoring, unless it is deemed medically emergent.^4,17^ In these cases, consideration of aerosol-generating procedures such as intubation or cautery should determine the appropriate PPE required and the safe distance from the patient.^2,4,17^ Only sterile single-use needle and corkscrew electrodes should be used. Surface electrodes should be single-use and discarded after use.^17^ Remote monitoring could reduce exposure to the neurophysiologist or technologist.^4,17^

## Future Directions (Table 4)

The COVID-19 pandemic has created opportunities for innovation and potential improvement in delivery of diagnostic testing and care for patients with neurological disorders. The remote clinical practice is now a global reality. Clinicians may consider providing some part of their electrophysiological care via phone or virtual platforms where internet or cellphone network is accessible. This could include screening of referrals, initial history taking, discussion of results with either patients or other health care personnel, seeking second opinions, team management meetings, and/or follow-up visits.^18,19^

**Table 4:** Future directions^18,19^

Clinicians may consider providing some part of the care via virtual platforms. This could include screening of referrals, initial history taking, discussion of results with either patients or other health care personnel, and/or follow-up visits. Development or revision of local guidelines on appropriate referral criteria for neurodiagnostic studies could help to optimize the use of clinical neurophysiology resources. Local guidelines could also address the need for and frequency of follow-up studies. Validation of the utility and the cost-effectiveness of remote–virtual care needs further assessment. These guidelines may be revised if and when more evidence become available.

The development or revision of local guidelines on appropriate referral criteria for neurodiagnostic studies could optimize the use of clinical neurophysiology resources, minimizing unnecessary testing and prioritizing studies that otherwise could have been delayed due to saturation of the health system. Local guidelines could also address the need for and frequency of follow-up studies in those cases where neurophysiological studies are the only objective way to measure improvement of medical or surgical interventions.^18,19^

Validation of the utility and the cost-effectiveness of remote–virtual care needs further assessment. The lack of reported data on the pros and cons of virtual care in neurophysiology in comparison to in-person visits merits further assessment in the field.

These guidelines may also serve as a useful reference in the case of future pandemics. Eventual resolution of the pandemic or the development of successful therapy for COVID-19 may make these guidelines obsolete. However, in the meantime, following the above recommendations is crucial to minimize the spreading of COVID-19. Revised versions will be made available on the CSCN website.
